# Cellular Retinoic Acid Binding Protein 2 Is Strikingly Downregulated in Human Esophageal Squamous Cell Carcinoma and Functions as a Tumor Suppressor

**DOI:** 10.1371/journal.pone.0148381

**Published:** 2016-02-03

**Authors:** Qingyuan Yang, Rui Wang, Weifan Xiao, Fenyong Sun, Hong Yuan, Qiuhui Pan

**Affiliations:** 1 Department of Clinical Laboratory Medicine, Tenth People’s Hospital of Tongji University, Shanghai, 200072, China; 2 Central Laboratory, Tenth People’s Hospital of Tongji University, Shanghai, 200072, China; 3 Department of Clinical Laboratory Medicine, The first affiliated hospital of Dalian Medical University, Tianjin, 300070, China; University of Saarland Medical School, GERMANY

## Abstract

Esophageal squamous cell carcinoma (ESCC) is the predominant pathotype of esophageal carcinoma (EC) in China, especially in Henan province, with poor prognosis and limited 5-year survival rate. Cellular retinoic acid binding protein 2 (CRABP2) is a member of the retinoic acid (RA) and lipocalin/cytosolic fatty-acid binding protein family and plays a completely contrary role in tumorigenesis through the retinoid signaling pathway, depending on the nuclear RA receptors (RAR) and PPARbeta/delta receptors. Presently, the biological role of CRABP2 in the development of ESCC has never been reported. Here, we firstly evaluated the expression of CRABP2 at both mRNA and protein levels and showed that it was remarkably downregulated in clinical ESCC tissues and closely correlated with the occurrence position, pathology, TNM stage, size, infiltration depth and cell differentiation of the tumor. Additionally, the biological function assays demonstrated that CRABP2 acted as a tumor suppressor in esophageal squamous carcinogenesis by significantly inhibiting cell growth, inducing cell apoptosis and blocking cell metastasis both *in vitro* and *in vivo*. All in all, our finding simplicate that CRABP2 is possibly an efficient molecular marker for diagnosing and predicting the development of ESCC.

## Introduction

Transcriptional activation of the nuclear receptor RAR by retinoic acid (RA), the signaling of which is frequently impaired during tumorigenesis, often leads to the inhibition of cell growth [1]. Cellular retinoic acid binding protein 2 (CRABP2) belongs to a family of small cytosolic lipid binding proteins, specific carriers for Vitamin A [2, 3], shuffling RA from cytoplasm into nucleus and forming a complex with nuclear RAR to facilitate the transcriptional activities of RA [4]. Previous studies support that CRABP2 is highly and specifically expressed in pancreatic ductal adenocarcinoma (PDAC) and more commonly expressed in high-grade precursor cancerous lesions than in low-grade lesions, which make it a specific diagnostic molecular marker to distinguish PDAC from other benign pancreatic conditions [5]. On the contrary, it has also been reported to directly interact with HuR to markedly increase its affinity for some target transcripts, enhancing their stability to facilitate its antioncogenic activity in mammary carcinoma cells [6]. Above all, it seems that CRABP2 displays oncogenic or anti-oncogenic activities in different tumors.

Esophageal carcinoma (EC) ranks sixth among all cancers in mortality in the world [7] and the fourth most common incidence cancer in China [8]. Henan province, a region in north central China, has the highest esophageal cancer rates in China and nearly all of these cases are esophageal squamous cell carcinoma (ESCC). Despite the tremendous progress in diagnosis and therapy, the prognosis of ESCC remains poor, for that the average 5-year overall survival rate is merely 40% [9]. Thus in this precision medical world, it is imperative to identify special and individual molecular biomarkers and targets associated with clinical phases of ESCC in order to diagnose the development of tumors, to predict the survival of patients, to evaluate the treatment efficiency and to guide the clinicians in therapy. To our present knowledge, the expression and biological functions of CRABP2 in ESCC has never been reported.

As a result, in the study, we firstly evaluated the expression of CRABP2 at both mRNA and protein levels in clinical ESCC tissues (T) and paired adjacent normal tissues (N), using the quantitative real-time PCR (qRT-PCR) and immunohistochemistry assays (IHC), respectively. Interestingly, both results showed that CRABP2 was remarkably and specifically downregulated in T tissues, compared with that in N tissues. Further analysis of the correlation of CRABP2 expression with clinical characterization showed that downregulation of CRABP2 was closely correlated with occurrence position, pathology, TNM stage, size, infiltration depth and cell differentiation of EC. Moreover, the following biofunctional assays showed that CRABP2 significantly inhibited cell growth, induced cell apoptosis, promoted G1/S checkpoint transition and blocked cell metastasis both *in vitro* and *in vivo*, implicating that CRABP2 is a possible candidate for the diagnosis, targeted therapy and prognosis of ESCC in the future.

## Materials and Methods

### Cell culture

EC109 cells obtained from American Type Culture Collection (ATCC) were maintained in the complete Dulbecco’s Modified Eagle’s Medium (DMEM) (HyClone), supplemented with 12% FBS (GIBCO), 100 units/ml penicillin and 100 μg/ml streptomycin (GIBCO), and were kept at 37°C in a humidified incubator with 5% CO2.

### Plasmids construction and transfection

The cDNA fragment of human CRABP2 was inserted between the Nhe I and Not I restriction enzyme sites of the lentivirus-mediated overexpression vector pGIPZa (Vector), using the primers 5’-CCGGGCTAGCCCCAACTTCTCTGGCAACTG-3’ (Forward, F) and 5’-AACGCGGCCGCTCACTCTCGGACGTAGACC-3’ (Reverse, R) (CRABP2-OE). The short hairpin RNAs against different sequences of CRABP2 (shCRABP2 #1, 5’- GAGGGAGACACTTTCTACA-3’; shCRABP2 #2, 5’- CCACAGAGATTAACTTCAA-3’) were constructed using the pLKO.1 vector (shCon.). All the above plasmids were confirmed by sequencing. To establish stable cell lines, the CRABP2 OE, shCRABP2 #1, #2 and the corresponding control plasmids were firstly packaged in 293T cells according to the guidelines of Lipofectamine 2000 (Invitrogen) and then infected EC109 cells for 72 h, followed by the selection with 1 μg/ml puromycin for 7 days.

### Quantitative real-time PCR(qPCR) assay

Total RNAs from paired esophageal tumor tissues (T) and the adjacent normal tissues (N) (n = 47), collected from patients who were diagnosed between January 2009 and December 2011 at the Department of Pathology, Anyang Tumor Hospital, Fourth Affiliated Hospital of Henan University of Science and Technology [10], were extracted using the Trizol reagent (Invitrogen), followed by the reverse transcription using the PrimeScriptTM reagent kit (TAKARA). Afterwards, the prepared cDNAs were subjected to qPCR on the 7900HT Fast (Applied biosystems) using the universal qPCR kit (KAPA Biosystems). The primers used in the study were summarized as follows: 18S rRNA (F: 5’-CCTGGATACCGCAGCTAGGA-3’, R: 5’-GCGGCGCAATACGAATGCCCC-3’), Vimentin (F: 5’-CCACTGAGTACCGGAGACA-3’, R: 5’-CGAAGGTGACGAGCCATT-3’) and CRABP2 (F: 5’-AGGAGCAGACTGTGGATGG-3’, R: 5’-AGTGAAGCAGGGCGGTGA-3’). The relative mRNA expression of Vimentin and CRABP2 were calculated using the 2^-ΔΔCt^ formula. All the samples were performed in triplicates and data were represented as mean ± STD from three independent experiments

### Cell growth analysis

About 3x10^3^ EC109 cells stably transfected with Vector or CRABP2 OE were seeded into the 96-well plates. Afterwards, the cell growth were measured at the indicated time points using the CCK-8 kit (DOJINDO, Japan), according to its instructions. Finally, the cell growth of Vector or CRABP2 OE cells at different days were normalized to the corresponding cell growth at Day 0, termed the relative cell growth.

### Colony formation assay

About 1x10^3^ EC109 cells stably transfected with Vector or CRABP2 OE were seeded into the 12-well plates in triplicate (#1, #2, #3) and left to grow in the incubator. 14 days later, the cells in the plates were fixed with 95% ethanol and then stained with 0.1% crystal violet (Sigma). Finally, the plates were pictured using the Canon digital camera.

### Hoechst staining assay

To evaluate the effects of CRABP2 on cell apoptosis, EC109 cells stably transfected with Vector or CRABP2 OE were seeded into the 24-well plates. The next day, the cells were washed with PBS and stained with Hoechst 33342 (KeyGen) according to the manufactures. At the end, the plates were pictured under the inverted fluorescence microscope (Leica, scale bar is 100 μm) and the cells with brilliant blue nucleus were defined as apoptotic cells.

### Flow cytometry assay

Stable EC109 cells transfected with Vector or CRABP2 OE were trypsined and stained with APC conjugated Annexin V and 7-AAD at room temperature. Afterwards, the percentage of apoptotic cells in about 10,000 cells were tested and analyzed using the flow cytometry (BD Biosciences). For the cell cycle analysis, stable EC109 cells transfected with Vector or CRABP2 OE were firstly starved for 12 h to be synchronized at G1 phase and then released for 24 h and 48 h, respectively, to analyze the cell cycle distribution. Data from three independent experiments were represented as mean ± STD, followed by the statistical analysis.

### Wound-healing assay

The cells stably transfected with Vector or CRABP2 OE were seeded into the 6-well plates and allowed to adhere overnight. The next day, the cells were scrached with sterile 200 μl tips, washed and cultured with fresh medium, supplemented with only 2% FBS. Afterwards, the wounds were pictured at the indicated time points to assess the cell migration (scale bar is 100 μm).

### Transwell assay

About 5x10^3^ EC109 cells stably transfected with Vector of CRABP2 OE were plated into the upper chambers (Corning) with serum-free medium. Simultaneously, the lower chambers (Corning) were filled with complete DMEM, supplemented with 12% FBS. 24 h later, the upper chambers were washed, fixed with 95% ethanol and stained with 0.1% crystal violet (Sigma). Finally, the cells migrated through the membrane to the lower surface of the upper chamber were pictured under the inverted microscope (Leica, scale bar is 100 μm).

### Western blotting assay

Total protein extracted from cell pellets or tissue samples were firstly quantified using the Pierce^®^ BCA Protein Assay Kit (Thermo Scientific) and then 80 μg protein were loaded on the SDS-PAGE gel for electrophoresis. Afterwards, the protein were transferred to the nitrocellulose membranes (NC, millipore), which were then blocked with 5% non-fat milk and incubated with the indicated primary antibodies, namely GAPDH (Proteintech, 60004-1-AP), β-actin (Sigma, A1978), Vimentin (Cell Signaling Technology, #5741), CRABP2 (Proteintech, 10225-1-AP), E-caderin (Abcam, ab76055), at 4°C overnight. The next day, the membranes were incubated with appropriate secondary antibodies conjugated with fluorescence [Licor, 926–32210 (Mouse) or 926-32211(Rabbit)]. Finally, the membranes were visualized using the Odyssey Infrared Imaging System (Licor).

### Immunohistochemistry (IHC)

The tissue microarrays (TMAs) composed of 100 pairs of esophageal tumor tissues (T) and adjacent normal tissues (N), the same set as our previously used TMAs [10], were dewaxed, hydrated and quenched the peroxidase activity in sequence. Afterwards, the slides were incubated with the antibody against CRABP2 at 4°C overnight. The next day, the slides were washed and incubated with the appropriate secondary antibody. Finally, the expression of CRABP2 in the N and T tissues was blindly evaluated by our colleague using the criteria that the staining of CRABP2 in epithelial EC tissues less than 5% was defined as negative (−), whereas more than 5% was positive (+). Representative images were taken under an inverted microscope (Leica, scale bar is 100 μm).

### Establishment of tumor-bearing nude mice

To evaluate the effects of CRABP2 on cell growth *in vivo*, 100 μl, 5x10^6^ EC109 cells stably transfected with Vector or CRABP2 OE were subcutaneously injected into the right side or the left side of the male nude mice (n = 5), respectively. All the mice aged 4 weeks were commercially purchased from Shanghai Super-B&K Laboratory Animal Corp. Ltd (Shanghai, China). The mice were then monitored every 2 days and the tumors were visible 10 days later, the day of which was recognized as day 0 for the tumor growth. Afterwards, the tumor sizes were measured every 5 days using a vernier caliper. The tumor volumes were calculated using the formula: length (mm) x width (mm) x width (mm) x 0.52, according to our previous reports [10]. 20 days later, the mice were firstly narcotized using 1% pentobarbital sodium and then kindly killed by breaking the neck to death. Finally, the tumors were dissected, weighed and pictured. It was noteworthy that no mice died during the experimental process and all the above animal experiments were carried out in the animal center of Shanghai Tenth Hospital of Tongji University, meeting with the criteria of SPF animal house and approved by the Animal Experiment Management Committee of Shanghai.

### Statistical analysis

The Student’s *t*-test and the ^2^ test were appropriately applied to assess the statistical significance in the study using the SPSS statistical 17.0. The gray values of western blotting bands were obtained using the WCIF ImageJ software. Error bars represent the standard deviation (STD) from three independent experiments. **p (two-tailed) < 0.01 and *p (two-tailed) < 0.05 were deemed with significance.

## Results

### CRABP2 is strikingly downregulated in esophageal tumor tissues

The differentiation-promoting RA chaperon protein CRABP2 has been reported to bind intracellular RA with high affinity and subsequently translocates to the nucleus, where it interacts with RA receptors and catalyzes RA-induced differentiation [11, 12]. Moreover, CRABP2, as a diagnostic and targeting biomarker, has been studied in a large number of carcinomas, for instance, the prostate cancer [13], the head and neck tumors [14], the primary retinoblastoma tumors [15], the non-small cell lung cancer [1] and the Wilms tumors[16]. However, the expression of CRABP2 in different tumors are quite different, leading to the dual roles in tumorigenesis.

**Fig 1:**
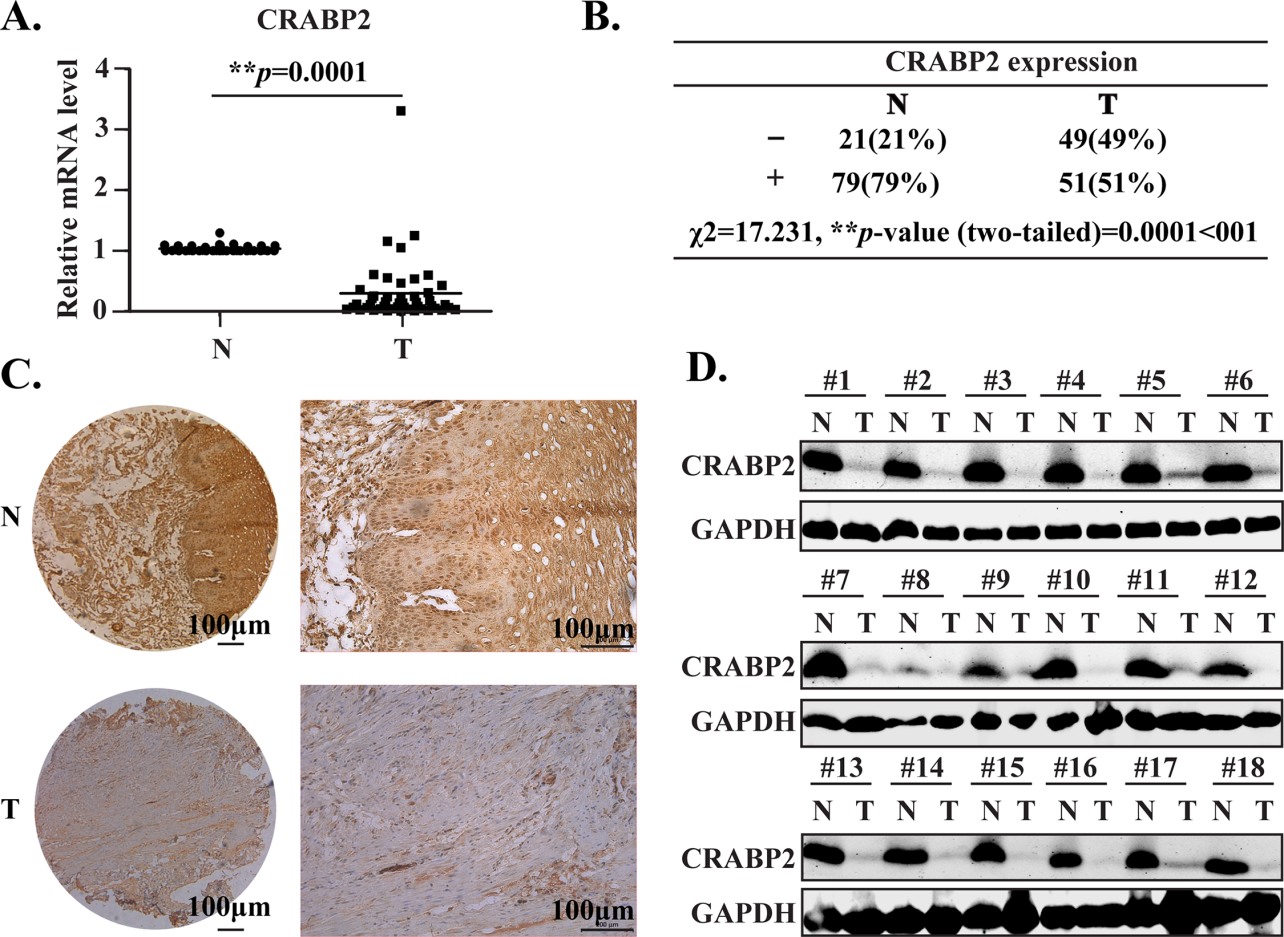
CRABP2 is strikingly downregulated in esophageal tumor tissues. **A.** The relative mRNA level of CRABP2 in paired esophageal tumor tissues (T) and adjacent normal tissues (N) was determined by RT-qPCR and was statistically analyzed using the paired two-tailed *t*-test (n = 47, **p = 0.0001). **B.**The ^2^ test was used to statistically analyze the CRABP2 protein expression in paired esophageal N and T tissues (n = 100, ^2^ value = 17.231, **p = 0.001).-, negative; +, positive.**C.** Representative immunohistochemistry (IHC) results of CRABP2 in esophageal N and T tissues. Scale bar is 100μm. **D.**Total protein from esophageal N and T tissues (n = 18) were extracted and subjected to western blotting assays to determine the expression of CRABP2, taking GAPDH as an internal reference.

To our present knowledge, there have been no data reporting the expression and biological roles of CRABP2 in esophageal tumorigenesis. Therefore, using the qPCR assays, we evaluated the relative mRNA expression of CRABP2 in clinical esophageal N and T tissues. Interestingly, in line with its expression pattern in neck and head tumors [14] and prostate cancer [13], CRABP2 was dramatically downregulated in the T tissues (Fig 1A, n = 47, **p = 0.0001<0.01). Moreover, taking advantage of TMAs, the same lot with those we previously used [10], we examined the protein expression of CRABP2 in esophageal N and T tissues using the IHC assays, the results of which were blindly evaluated by another colleague. Data from IHC were statistically analyzed using the ^2^ test. As shown in Fig 1B, we found that the CRABP2 protein were strikingly downregulated in the T tissues (^2^ value = 17.231, **p = 0.0001<0.01). Additionally, the representative IHC results in N and T were shown in Fig 1C. Furthermore, to confirm the above IHC results, the total protein extracted from N and T tissues (n = 18) were subjected to western blotting assays. We found that CRABP2 protein in esophageal T tissues were dramatically downregulated (Fig 1D), consistent with the gene expression profiling data performed by Uchikado. et al that CRABP2 is significantly downregulated in the tumor tissues [17]. Above all, we concluded that CRABP2 at both mRNA and protein levels were significantly downregulated in ESCC tissues, compared to the adjacent N tissues.

### Clinical characterization of CRABP2 in esophageal tissues

Subsequently, we analyzed the clinical characterization of CRABP2 expression in esophageal N and T tissues using the ^2^ test. As demonstrated in Table 1, CRABP2 was greatly downregulated in the T tissues independent of age (**p = 0.018 for < 60 years old and **p = 0.001 for ≥ 60 years old), gender (**p = 0.005 for male and **p = 0.002 for female), and the lymphatic metastasis (**p = 0.004 for negative and **p = 0.002 for positive). However, the downregulation of CRABP2 in T tissues was closely correlated with the position of tumor (**p = 0.001 for middle position), the gross pathology (**p = 0.0001 for Ulcerative pathology), the TNM stage (**p = 0.003 for stage II and *p = 0.013 for stage III), the tumor size (*p = 0.014 for ≥ 10 mm^3^, ≤ 20 mm^3^, **p = 0.0001 for >20 mm^3^), the infiltration depth (*p = 0.034 for muscularis and **p = 0.001 for fibrosa), and the cell differentiation (*p = 0.011 for moderately differentiated tumors and *p = 0.021 for well differentiated tumors) as well. Consequently, we speculated that the downregulation of CRABP2 predicted the poor development of ESCC.

**Table 1:** Clinical characterization of CRABP2 expression in paired esophageal tumor tissues and adjacent normal tissues.

	Expression of CRABP2 protein					
	N		T			
	-	+	-	+	χ^2^	*P*-value
**Age(years)**						
<60	6(17.6%)	28(82.4%)	15(44.1%)	19(55.9%)	5.581	0.018
> = 60	14(22.6%)	48(77.4%)	32(51.6%)	30(48.4%)	11.197	0.001
**Gender**						
Male	14(22.6%)	48(77.4%)	29(46.8%)	33(53.2%)	8.01	0.005
Female	6(17.6%)	28(82.4%)	18(52.9%)	16(47.1%)	9.273	0.002
**Position of tumor**						
Upper	2(18.2%)	9(81.8%)	6 (54.5%)	5(45.5%)	3.134	0.076
Middle	13(21.7%)	47(78.3%)	30(50%)	30(50%)	10.474	0.001
**Gross pathology**						
Medullary	13(33.3%)	26(66.7%)	20(51.3%)_	19(48.7%)	2.574	0.109
Fungating	0(0%)	4(100%)	2(50%)	2(50%)	2.667	0.102
Ulcerative	4(8.9%)	41(91.1%)	21(46.7%)	24(53.3%)	16.006	0.0001
**TNM stage**						
I	3(60%)	2(40%)	4(80%)	1(20%)	0.476	0.49
II	7(16.3%)	36(83.7%)	20(46.5%)	23(53.5%)	9.124	0.003
III	10(23.3%)	33(76.7%)	21(48.8%)	22(51.2%)	6.103	0.013
**Tumor size (cm**^**3**^**)**						
<10	10(28.6%)	25(71.4%)	14(40%)	21(60%)	1.014	0.314
> = 10,< = 20	7(18.4%)	31(81.6%)	17(44.7%)	21(55.3%)	6.09	0.014
>20	3(13%)	20(87%)	16(69.6%)	7(30.4%)	15.154	0.0001
**Infiltration depth**						
Submucosa	1(20%)	4(80%)	2(40%)	3(60%)	0.476	0.49
Muscularis	3(16.7%)	15(83.3%)	9(50%)	9(50%)	4.5	0.034
Fibrosa	16(23.5%)	52(76.5%)	34(50%)	34(50%)	10.247	0.001
**Lymphatic metastasis**						
Negative	16(29.6%)	38(70.4%)	31(57.4%)	23(42.6%)	8.476	0.004
Positive	4(9.5%)	38(90.5%)	16(38.1%)	26(61.9%)	9.45	0.002
**Differentiation**						
Undifferentiated	1(25%)	3(75%)	3(75%)	1(25%)	2	0.157
Poorly differentiated	3(21.4%)	11(70.6%)	8(57.1%)	6(42.9%)	3.743	0.053
Moderately differentiated	4(12.1%)	29(87.9%)	13(39.4%)	20(60.6%)	6.418	0.011
Well differentiated	13(27.1%)	35(72.9%)	24(50%)	24(50%)	5.321	0.021

### CRABP2 remarkably inhibits cell growth, induces apoptotic cell death and promotes G1/S checkpoint transition

**Fig 2:**
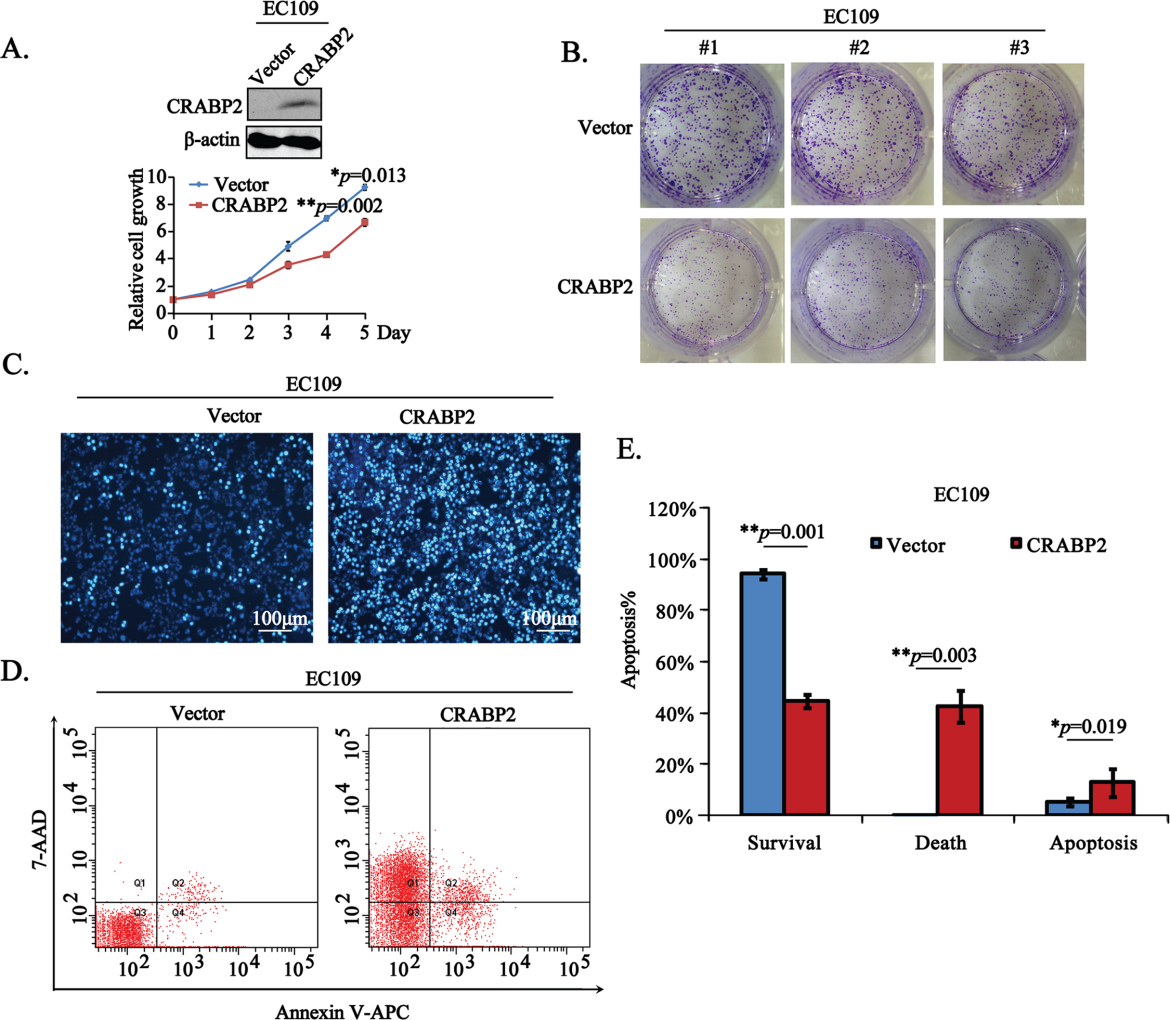
CRABP2 inhibits cell growth and induces apoptotic cell death in esophageal tumor cells. **A.** The cell growth of EC109 cells stably transfected with Vector or CRABP2 OE were examined using CCK8 at the indicated days, which was normalized with the corresponding cell growth of day 0, termed the relative cell growth (**p = 0.002 for day 4 and *p = 0.013 for day 5). The stable CRABP2 overexpression in EC109 cells were confirmed using the Western blotting assays. **B.** EC109 cells stably transfected with Vector or CRABP2 OE were subjected to colony formation assays in triplicate (#1, #2, #3). **C.** EC109 cells stably transfected with Vector or CRABP2 OE were stained with Hoechst 33342.The representative pictures were taken under the inverted microscope. Scale bar is 100 μm. **D-E.** EC109 cells stably transfected with Vector or CRABP2 OE were subjected to flow cytometry to test the cell apoptosis (D). Data from three independent experiments were statistically analyzed (E, **p = 0.001 for survival, **p = 0.003 for death and *p = 0.019 for apoptosis).

In order to explore the biological roles of CRABP2 in esophageal tumorigenesis, we established stable EC109 cells transfected with Vector or CRABP2 OE plasmids. Using the CCK8 kit, we measured the effects of upregulated CRABP2 on cell proliferation. Interestingly, we found that upregulation of CRABP2 significantly inhibited cell proliferation, compared with cells transfected with Vector (Fig 2A, **p = 0.002 for day 4 and *p = 0.013 for day 5). Moreover, we performed the colony formation assays to confirm the negative role of CRABP2 in cell growth. In line with above results, the triplicate data from colony formation assays showed that CRABP2 indeed remarkably suppressed the esophageal tumor cell growth (Fig 2B).

Subsequently, we analyzed the effects of CRABP2 on cell apoptosis using the Hoechst 33342 staining assay [18] and the results displayed that the cell apoptosis was obviously increased in CRABP2 OE transfected EC109 cells (Fig 2C). In addition, the results from flow cytometry assays showed that the survival of EC109 cells were apparently more in Vector transfected cells than CRABP2 OE cells (**p = 0.001), whereas the percentage of cell death (**p = 0.003) and apoptosis (*p = 0.019) were greatly higher in CRABP2 OE cells (Fig 2D and 2E).

**Fig 3:**
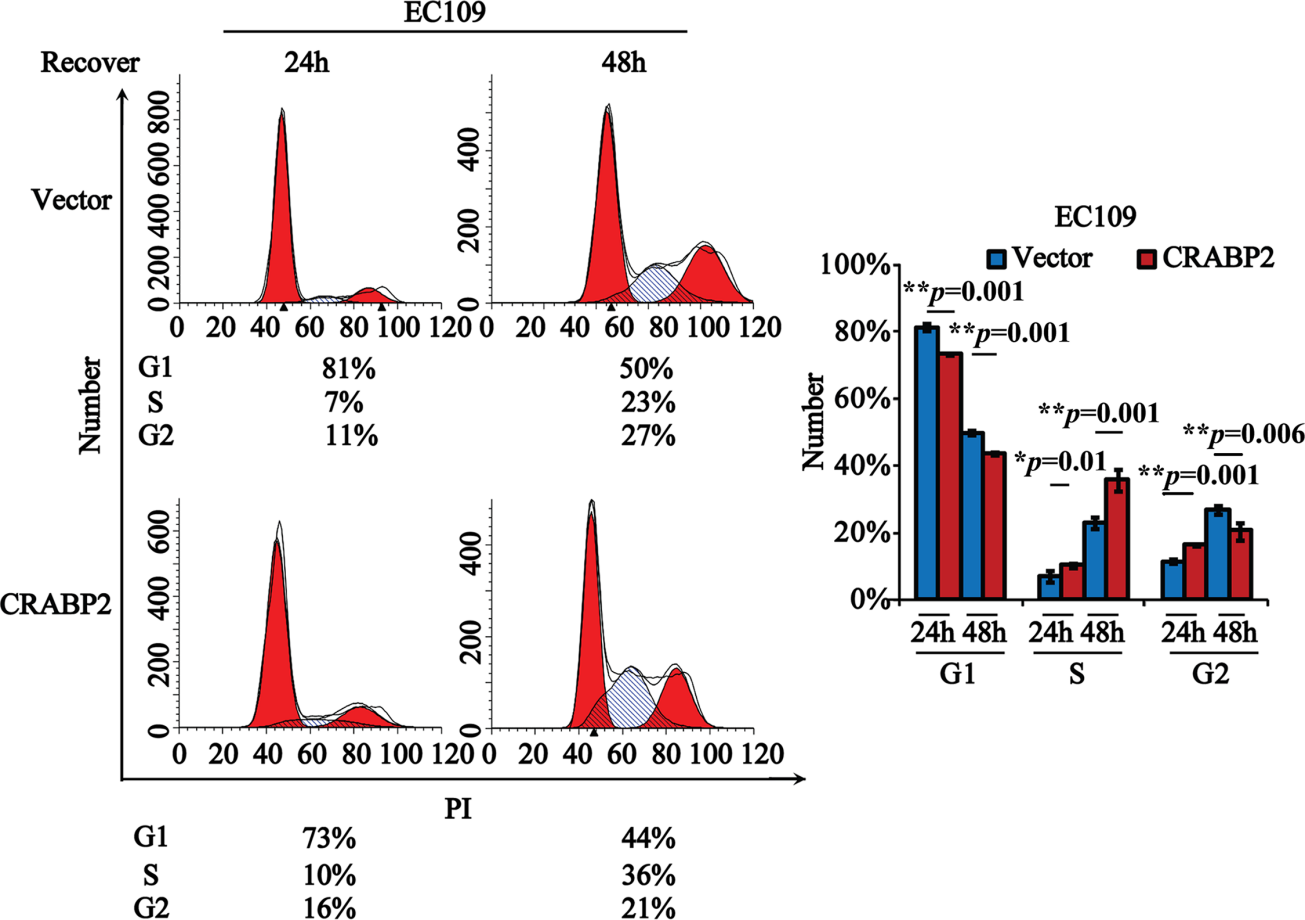
CRABP2 promotes G1/S checkpoint transition in esophageal tumor cells. Stable EC109 cells transfected with Vector or CRABP2 OE were synchronized by starvation for 12 h and then released to be further cultured in the incubator for 24 h and 48 h, respectively, followed by the subjection to the flow cytometry analysis. Data from three independent experiments were statistically analyzed and graphically depicted (**p = 0.001 for G1 phase at both 24 h and 48 h, *p = 0.01 and **p = 0.001 for S phase at 24h and 48 h, respectively, **p = 0.001 and **p = 0.006 for G2 phase at 24 h and 48 h, respectively)

Furthermore, we investigated the biological functions of CRABP2 in cell cycle. As shown in Fig 3, the G1 phase of CRABP2 OE transfected stable EC109 cells was significantly less than that of Vector transfectedEC109 cells, whereas the S phase of CRABP2 OE transfected EC109 cells was much more than that of Vector transfected EC109 cells at both 24 h and 48 h, suggesting that CRABP2 might promote the G1/S checkpoint progression in esophageal tumor cells. Taken together, we considered that CRABP2 might act as a tumor suppressor in esophageal tumorigenesis.

### CRABP2 negatively regulates cell migration via epithelial-mesenchymal transition in esophageal cancer cells

**Fig 4:**
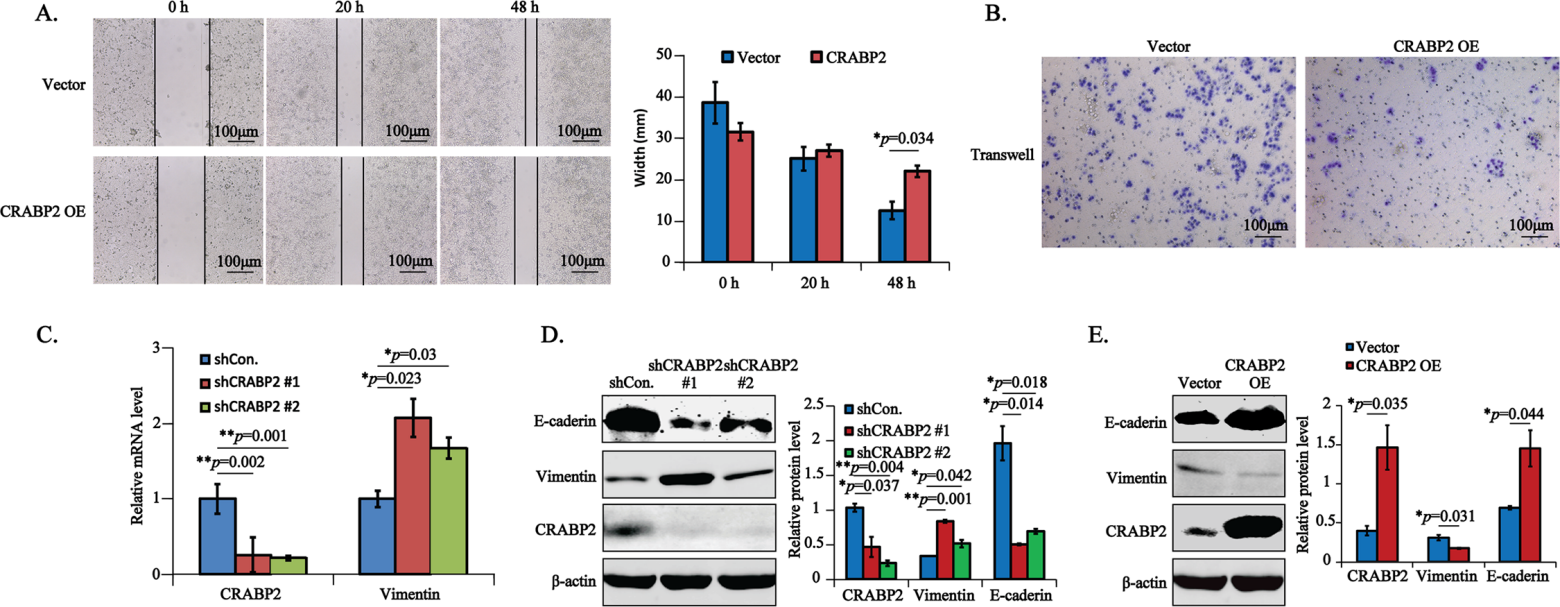
CRABP2 blocks cell metastasis via EMT. **A-B.** EC109 cells stably transfected with Vector or CRABP2 OE were subjected to wound-healing assays(A) and transwell assays (B). Data from three independent wound-healing experiments were represented as mean ± STD, followed by the two-tailed Student's t-test. Representative pictures were taken under the inverted microscope at the indicated time points. Scale bar is 100 μm.**C.**EC109 cells stably knockdown of CRABP2 using shRNA (shCRABP2 #1, #2) were subjected to RT-qPCR to examine the relative mRNA level of CRABP2 (**p = 0.002 for #1, **p = 0.001 for #2) and Vimentin (*p = 0023 for #1, *p = 0.03 for #2).**D-E.** EC109 cells downregulated (D, shCRABP2 #1, #2) or overexpressed (E, CRABP2 OE) of CRABP2 were subjected to western blotting assays to test the expression of CRABP2,Vimentin and E-caderin, taking β-actin as an internal reference. The gray values of bands from three independent experiments were subjected to the two-tailed statistical analysis.

Afterwards, we investigated the roles of CRABP2 in cell migration. Firstly, the wound-healing assays were used to explore the cell migration in Vector and CRABP2 OE stably transfected EC109 cells. Interestingly, consistent with its antioncogenic activities, the overexpressed CRABP2 apparently blocked cell migration at 48 h (Fig 4A, *p = 0.034). Afterwards, the transwell assays were used to confirm the above results. As shown in Fig 4B, CRABP2 OE transfected EC109 cells migrated to the membrane of the upper chambers were strikingly less than that of Vector transfected cells. Notably, to our knowledge, we are the first team reporting the role of CRABP2 in cell metastasis. It has been reported that epithelial-mesenchymal transition (EMT) is an essential step for cancer metastasis, which is characterized by loss of epithelial markers like E-caderin and increase of mesenchymal markers such as Vimentin [19]. Therefore, to determine whether the EMT participated in CRABP2 induced cell migration, we analyzed the expression of Vimentin and E-caderin using the qPCR and/or western blotting assays. In line with above biological assays, when the CRABP2 was strikingly downregulated (**p = 0.002 for shCRABP2 #1 and **p = 0.001 for shCRABP2 #2), the relative mRNA expression of Vimentin was remarkably increased (Fig 4C, *p = 0.023 and *p = 0.03 for shCRABP2 #1, #2, respectively). Furthermore, the Vimentin protein was significantly upregulated (**p = 0.001 and *p = 0.042, respectively), accompanied by the downregulation of E-caderin (*p = 0.014 and *p = 0.018, respectively) in CRABP2 knockdown cells (Fig 4D). In addition, Vimentin was reduced and E-caderin was increased in CRABP2 OE cells (Fig 4E, *p = 0.031 and *p = 0.044, respectively). These results indicated that CRABP2 negatively regulated cell metastasis by modulating EMT in esophageal tumor cells.

### CRABP2 inhibits tumor growth *in vivo*

**Fig 5:**
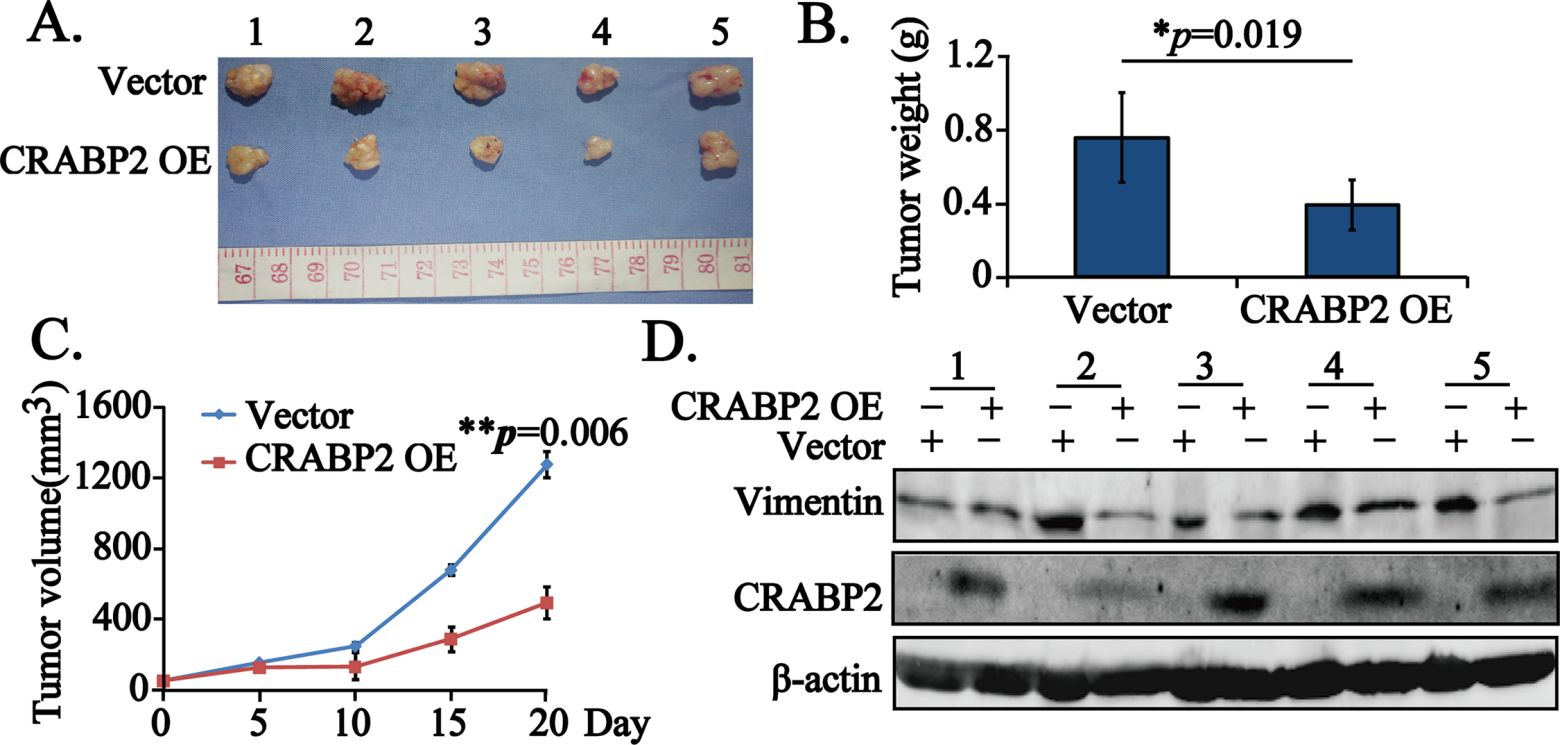
CRABP2 significantly inhibits esophageal tumor cell growth *in vivo*. **A.** The tumors dissected from the tumor-bearing nude mice(n = 5), which were subcutaneously injected with Vector or CRABP2 OE transfected EC109 cells. **B.** The weight of tumors in A was represented as mean ± STD and statistically analyzed (*p = 0.019). **C.** The volumes of tumors in A were graphically depicted and statistically analyzed at the indicated days (**p = 0.006 for day 20). **D.** Total protein extracted from tumor tissues in A were subjected to western blotting assays to confirm the overexpression of CRABP2 and to examine the expression of Vimentin, taking β-actin as an internal reference.

Taken the above *in vitro* data into consideration, we thought that CRABP2 acted as a tumor suppressor by inhibiting cell growth, inducing cell apoptosis and suppressing cell migration via EMT. To further consolidate its antioncogenic role *in vivo*, we established the tumor-bearing nude mice model by subcutaneously injecting the stably transfected Vector and CRABP2 OE EC109 cells. At the terminal day, the mice were kindly put to death and the tumors were dissected and pictured (Fig 5A). Afterwards, the tumors were weighed and data were shown as mean ± STD. Remarkably, the weight of CRABP2 OE tumors was less than that of Vector tumors (Fig 5B, *p = 0.019). Consistent with the *in vitro* cell growth inhibition, the volumes of CRABP2 OE tumors along the experiments were significantly smaller than those of Vector tumors (Fig 5C, **p = 0.006), confirming that CRABP2 suppressed the cell growth.

As we had shown that CRABP2 regulated cell migration via EMT *in vitro*, we further examined the expression of Vimentin in CRABP2 OE tumor tissues dissected from the nude mice, in comparison with that in Vector tumor tissues. Surprisingly, we found that the expression of Vimentin was strikingly reduced in five of CRABP2 OE tumor tissues (Fig 5D), suggesting that CRABP2 was of great possibility to regulate cell metastasis by inhibiting Vimentin expression *in vivo*.

## Discussion

Previous studies have demonstrated that CRABP2 is epigenetically downregulated in a large number of carcinomas, such as prostate cancer [13], human head and neck tumors [14], astrocytic gliomas [20]. On the contrary, some other investigators have shown that CRABP2 is highly upregulated and correlated with poor outcome in primary retinoblastoma tumors [15], non-small cell lung cancer [1], advanced breast cancer [21] and Wilms tumors [16].Therefore, the roles of CRABP2 in tumorigenesis, depending on the genetic background and the tumor pathotype, are more complicated than we have expected. To our knowledge, there have been no reports of CRABP2, especially its biological functions, in EC, which is one of the least studied and deadliest cancers worldwide due to its extremely aggressive nature and poor survival rate [22]. Moreover, the ESCC is the most predominant pathotype of EC in China and caused up to 11.2% of all cancer deaths and ranked as the fourth most common cause of cancer mortality [23]. Therefore, in the precision medical environment, to identify specific molecular biomarkers for predicting the development of ESCC is of great importance for our Chinese population. In the study, we have provided sufficient evidence that CRABP2 acts as a tumor suppressor in esophageal squamous tumorigenesis. Firstly, it is strikingly downregulated in clinical esophageal squamous tumor tissues both at mRNA and protein levels. Secondly, it significantly inhibits esophageal tumor cell growth both *in vitro* and *in vivo*, and induces apoptotic cell death. Thirdly, CRABP2 negatively regulates cell metastasis of esophageal tumor cells via EMT.

First of all, data from qPCR, IHC and western blotting assays of paired clinical ESCC tissues, collected from patients who were diagnosed between January 2009 and December 2011 at the Department of Pathology, Anyang Tumor Hospital, Fourth Affiliated Hospital of Henan University of Science and Technology [10], revealed that the expression of CRABP2 was strikingly downregulated at both mRNA and protein levels. Subsequently, the clinical characterization of CRABP2 in ESCC tissues showed that downregulation of CRABP2 in T tissues was significantly correlated with the occurrence position, pathology, TNM stage, size, infiltration depth and cell differentiation of ESCC, suggesting that the expression of CRABP2 was likely to be an efficient indicator for the prognosis of ESCC. In head and neck squamous cell carcinoma, CRABP2 has been demonstrated to be highly methylated in the promoter region ranged from -450 to -117 [14]. Moreover, Benito Campos *et al*. has detected extensive CpG methylation upstream of the CRABP2 gene locus in a study sample comprising 100 astrocytic gliomas of WHO Grade II to IV, which is negatively correlated with CRABP2 mRNA expression [20]. However, whether the downregulation of CRABP2 in ESCC was attributed to the epigenetical methylation of its promoter were required to be further studied.

CRABP2 is a cytosolic-to-nuclear shuttling protein, which facilitates RA binding to its cognate receptor in the nucleus and functions in the retinoid signaling pathway [24, 25]. It has been reported to be epigenetically modulated by the trascription factors MyoD and Sp1 to promote myoblast differentiation in C2C12 cells [2]. Moreover, it has been shown to directly interact with RNA-binding protein HuR to enhance the stability of Apaf-1, a major protein in the apotosome, leading to the suppression of tumor cell proliferation [6]. In the study, using the CCK8 kit and the colony formation assays, we proved that CRABP2 remarkably inhibited the cell growth of esophageal tumor cells *in vitro*. The subsequent Hoechst 33342 staining and flow cytometry assays showed that CRABP2 could obviously induced apoptotic cell death of esophageal tumor cells. Additionally, further tumor-bearing nude mice experiments confirmed that CRABP2 in esophageal tumor cells could significantly suppress the cell proliferation *in vivo*. Thus, CRABP2 acted as a tumor suppressor in esophageal squamous tumorigenesis.

It is reported that high CRABP2 levels can make PDAC cells sensitive to ATRA-mediated growth inhibition and apoptosis with increased migration and invasion phenotypes [26], implicating that CRABP2 plays a functional role in cell metastasis. Therefore, in the study, we evaluated the role of CRABP2 in cell metastasis in esophageal tumor cells. Data from wound-healing and transwell assays showed that overexpression of CRABP2 greatly blocked esophageal tumor cell metastasis. And further western blotting examination of Vimentin, the EMT biomarker, in CRABP2 OE transfected EC109 cells *in vitro* and *in vivo* demonstrated that CRABP2 exerted its functions in cell metastasis by regulating EMT process in ESCC. Taken together, CRABP2 in esophageal tumorigenesis acted as a tumor suppressor not only by inhibiting cell proliferation, but also by blocking cell metastasis via EMT process.

The corresponding retinoid binding proteins CRABP2 and FABP5 have been shown to be critical intracellular partitioning factors of RA between the nuclear receptors RAR and PPARbeta/delta for either anti-survival or pro-survival effects [27]. As a result, we speculated that CRABP2 acted as a tumor suppressor in esophageal tumor tissues due to the high CRABP2/FABP5 ratio. However, the expression dependence of CRABP2 and FABP5 in EC tissues and the detailed antioncogenic mechanisms of CRABP2 in EC tumorigenesis remained to be further studied.

All in all, we firstly provide evidence that CRABP2 is strikingly downregulated in human ESCC, which is closely correlated with occurrence position, pathology, TNM stage, size, infiltration depth and cell differentiation of ESCC, and acts as a tumor suppressor both *in vitro* and *in vivo*, giving clues for further studies on the mechanisms of CRABP2 in esophageal tumorigenesis and providing a possible molecular candidate for predicting the development of EC.
